# Scorpion Envenomation of Lactating Rats Decreases the Seizure Threshold in Offspring

**DOI:** 10.3390/toxins13120853

**Published:** 2021-11-30

**Authors:** Marina de Oliveira Rodrigues Barbosa, Maria Eliza F. do Val de Paulo, Ana Leonor Abrahão Nencioni

**Affiliations:** Laboratory of Pharmacology, Butantan Institute, Av. Dr. Vital Brazil 1500, São Paulo 05503-900, Brazil; orb.marina@gmail.com (M.d.O.R.B.); maria.paulo@butantan.gov.br (M.E.F.d.V.d.P.)

**Keywords:** scorpion accidents, lactation, maternal care, seizure threshold

## Abstract

Few data are available in the literature describing the long-term effects of envenoming in the perinatal period. In this study, the relationship between envenoming of lactating rats and possible behavioral changes in the mother and in her offspring were investigated. Lactating Wistar rats received a single dose of *T. serrulatus* crude venom on postnatal days 2 (V2), 10 (V10) or 16 (V16), and had their maternal behavior evaluated. The seizure threshold was evaluated in adulthood offspring. A decrease in maternal care during envenoming was observed in V2 and V10 groups. The retrieval behavior was absent in the V2 group, and a lower seizure threshold in the adult offspring of all groups was observed. During envenoming, mothers stayed away from their offspring for a relatively long time. Maternal deprivation during the early postnatal period is one of the most potent stressors for pups and could be responsible, at least in part, for the decrease in the convulsive threshold of the offspring since stress is pointed to as a risk factor for epileptogenesis. Furthermore, the scorpionic accident generates an intense immune response, and inflammation in neonates increases the susceptibility to seizures in adulthood. Therefore, maternal envenoming during lactation can have adverse effects on offspring in adulthood.

## 1. Introduction

Scorpions are terrestrial arthropods, which inhabit different biomes and are distributed worldwide, except in Antarctica [1].

Scorpionism is the main cause of accidents with venomous animals in Brazil having overcome snakebite since 2004 [2]. *Tityus serrulatus*, popularly known as yellow scorpion, has been described in 1922 by Lutz and Mello and is responsible for most of the serious accidents with scorpions in Brazil [3,4,5].

Scorpion accidents are classified according to symptoms into: mild, characterized by local signs such as edema, erythema, sweating, numbness and twitching; moderate, in which, in addition to the previous symptoms, vomiting, abdominal pain, tachypnea, tachycardia or bradycardia, hypertension, agitation, hypersalivation and priapism can also occur; and severe, in which the main symptoms are cardiovascular and pulmonary complications such as heart failure and pulmonary edema, and neurological symptoms such as encephalopathy, coma and convulsion [6].

The number of scorpion accidents has increased in the last few decades and most of the victims consist of people of reproductive age, so pregnant and lactating women are becoming possible targets [4,5]. In the state of São Paulo, between 2007 and 2019, pregnant women comprised 3% of female victims of scorpionism, with the majority during the second trimester of gestation [7]. However, there are no data on the number of lactating victims, and it is difficult to estimate the real proportion of victims in the perinatal period.

Experimentally, some effects of pre- or post-natal injection of Brazilian scorpion venoms in rats have been demonstrated. Prenatal injection of *T. serrulatus* venom in mothers increased the number of post-implantation losses, altered some reflex and physical parameters of the pups, and caused an increase in the weight of the placentas, liver and lungs of the pups [8,9,10]. Prenatal injection of *T. bahiensis* venom also increased the weight of some organs in the pups and altered physical and behavioral parameters both in childhood and in adulthood [11,12,13] and, when injected during lactation, it caused a delay in physical and reflex development in childhood, and reduced anxiety in adulthood [14].

Pregnancy and breastfeeding are very important for the adequate development of the pups, both from a physical and a behavioral point of view. Particularly, the quality of care offered to the newborns is important for the maturation of cerebral architecture, especially the hippocampal areas responsible for cognitive functions and stress responsiveness [15,16,17]. Clinical studies have demonstrated that adverse conditions such as stress early in life predispose the individual to developing several psychiatric disorders such as anxiety, depression, and epilepsy [18,19,20]. Experimentally, a relationship was observed between stress in perinatal period and a decrease in neurogenesis [21,22]. In addition, stress is relevant to the process of epileptogenesis, both in childhood and in adult life [23]. Hormones and neurotransmitters mediating the influence of early-life stress on excitability may create a permanent vulnerability for the development of epilepsy [20].

It is of utmost importance that the short and long term effects of scorpionism during pregnancy and lactation are well elucidated, in order to minimize the damage to the health of affected mothers and children.

Previous empirical observations conducted in our laboratory revealed a change in the pattern of care provided by mothers envenomed by scorpions during breastfeeding.

Maternal care is responsible for the proper development of the central nervous system [15,17]. Therefore, it is necessary to investigate whether the correct development of the offspring is affected by the envenomation of the mothers. We believe that the stress caused by a single dose of scorpion venom can cause changes in the behavior of the mothers, resulting directly or indirectly in altered development of the offspring.

Therefore, the present study aimed to evaluate the effect of moderate maternal envenoming on the development of the offspring’s central nervous system, with particular attention paid to the susceptibility to seizures.

Pentylenetetrazole (PTZ), widely used as seizure-inducing drug, is a GABA_A_ antagonist and suppresses the function of inhibitory synapses, leading to increased neuronal activity and consequently causes generalized seizures in animals [24]. In small doses, PTZ has been used as a model for absence seizures and in higher doses it produces convulsive seizures [25]. Here, it was used to test the convulsive threshold of the offspring of envenomed rats.

## 2. Results

### 2.1. Maternal Behavior

**Figure 1:**
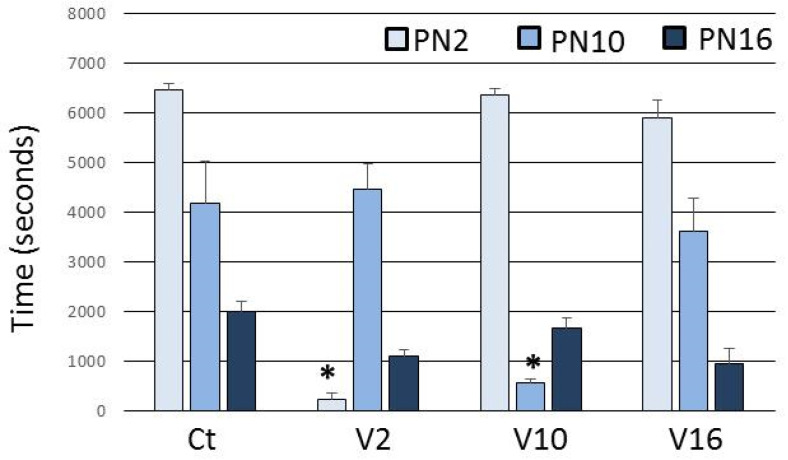
Time spent in arched-back nursing posture after the treatment. Ct injected with 0.9% NaCl in PN2, PN10 and PN16. V2 injected with *T. serrulatus* venom in PN2 and 0.9% NaCl in PN10 and PN16. V10 injected with *T. serrulatus* venom in PN10 and 0.9% NaCl in PN2 and PN16. V16 injected with *T. serrulatus* venom in PN16 and 0.9% NaCl in PN2 and PN10. (*n* = 6 females per group). Data are expressed as means ± SEM. (*) *p* < 0.001 compared to the control group (Two-way ANOVA).

The mothers in the Ct group spent most of the time in an arched-back nursing posture and the care offered to the pups decreased over the days (Figure 1). In the V2 and V10 groups, the time spent in the arched-back nursing posture is significantly reduced on the day of envenoming (Figure 1). In the V16 group, there was no change, because at this stage this behavior is almost extinguished (Figure 1).

### 2.2. Retrieval Test

The test was performed only on PN2 because, over the first week postpartum, the pups are able to move around and the mother ceases to exhibit the behavior of picking them up, and the frequency of licking/grooming and arched-back nursing decreases [15,26].

**Figure 2:**
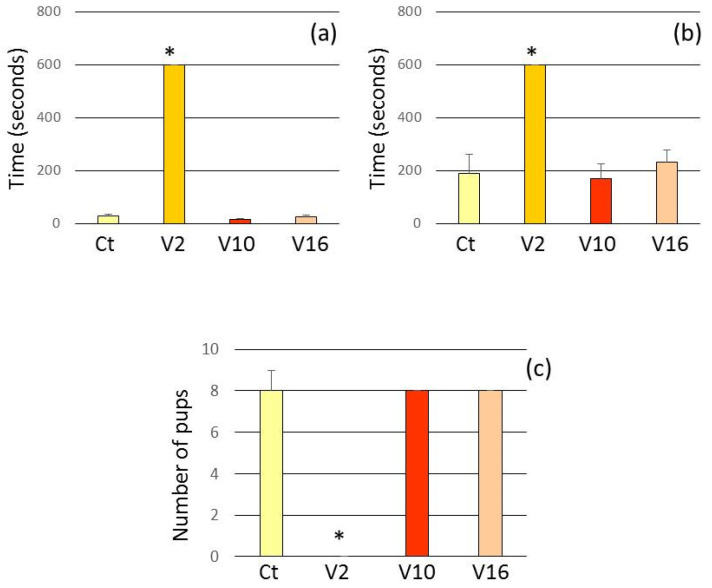
Assessment of mothers in retrieval test after the treatment. Ct injected with 0.9% NaCl in PN2, PN10 and PN16. V2 injected with *T. serrulatus* venom in PN2 and 0.9% NaCl in PN10 and PN16. V10 injected with *T. serrulatus* venom in PN10 and 0.9% NaCl in PN2 and PN16. V16 injected with *T. serrulatus* venom in PN16 and 0.9% NaCl in PN2 and PN10. (*n* = 6 females per group). (**a**) Latency to retrieve the first pup. (**b**) Latency to retrieve the last pup. (**c**) Number of retrieved pups. Data are expressed as means ± SEM. (*) *p* < 0.001 compared to the control group (Two-way ANOVA).

In the V2 group, dams that failed to group the litter during the test period demonstrated higher latencies to retrieve pups. In V10 and V16 groups, the mothers behaved similarly to the control group (Figure 2).

### 2.3. Seizure Threshold

**Figure 3:**
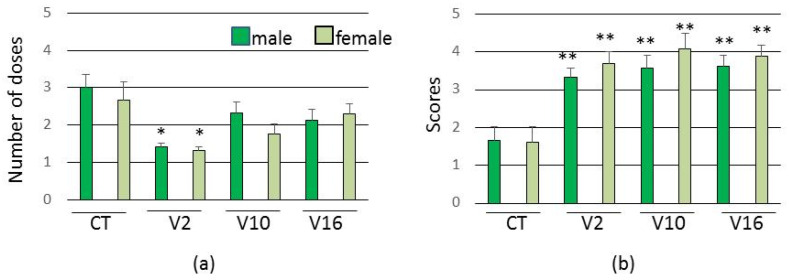
Assessment of seizure threshold (*n* = 6 females and 6 males per group). (**a**) Number of doses needed to reach the seizure threshold. (**b**) Average intensity of seizure behavior. Data are expressed as means ± SEM. (*) *p* < 0.01 and (**) *p* < 0.05 compared to the control group (Kruskal Wallis test).

In all groups, females and males obtained similar results, with no gender distinction. The V2 group developed seizure behavior earlier in relation to the control group, requiring fewer booster doses (Figure 3).

In addition, in all experimental groups the animals showed more intense convulsions than in the control group (Figure 3).

## 3. Discussion

Despite the increase in the number of scorpion accidents in the last few years, there is insufficient information on the effects of scorpion envenomation on pregnancy, lactation, and neonatal outcomes, and unfortunately the available data are controversial.

Experimental studies provide clues as to what might happen in an accident in humans. Thus, in the present study, we used lactating rats to evaluate the possible consequences for the offspring after a scorpion sting in the mother during breastfeeding.

A possible scorpion accident was simulated through a single subcutaneous injection of *T. serrulatus* venom, which is the most common method of inoculation in accidental bites. The dose of 4.0 mg/kg was determined in previous experiments in our laboratory (unpublished data) as causing symptoms observed in moderate to severe envenoming cases such as severe local pain, piloerection, respiratory perturbation and increased lacrimal and salivary secretions. The days of injection were chosen based on the different periods of brain maturation [27]. The development of the mammalian brain begins in embryogenesis, and its maturation continues in the postnatal period [27,28]. In rats, the neurogenesis in cortical regions starts on the ninth day of gestation and extends until the fifteenth postnatal day. The growth spurt of the brain corresponds to the period in which this organ increases in weight most rapidly. In rodents this event peaks around postnatal day 7, reviewed by [29].

Our results demonstrated a decrease in maternal care on the days of the venom injection, and offspring that were more susceptible to convulsion in adulthood. It is relevant to note that the earlier the envenoming, the lower the seizure threshold in the offspring.

In mammals, maternal care is the care that the mother provides to the offspring, and it is essential for the adequate development of the nervous system [15,17,30]. Maternal care modulates the expression of various genes and neurochemical content in one or more regions of the brain and changes in this process can cause variations in behavioral responses and the development of mood disorders [17,31].

Studies in animals and humans have shown that, during early childhood, the brain is particularly sensitive to stress [32], probably because in this period the system is still developing [19].

Maternal deprivation during the early postnatal period is one of the most potent stressors for pups [32] and it was experimentally demonstrated that it can affect behavior, ACTH and neurotrophin levels in rats, which persist into adulthood [33]. A stressful status can lead to permanent neurobehavioral alterations and an increased susceptibility to psychiatric disorders [34].

Epidemiological studies point to stress as a risk factor for epileptogenesis in adults and young people [20,35]. Experimentally, it was demonstrated that early-life stress in rats has long-lasting effects on brain excitability and may promote age-specific seizures and epilepsy [36]. On the other hand, increased maternal care makes mice genetically predisposed to epilepsy less susceptible to seizures [37].

In our experiments, we observed that mothers from groups V2, V10 and V16 remained away from the pups for a long period after venom injection. This is probably due to the fact that they were experiencing the symptoms of envenoming, mainly pain, as we could see from the vocalization that occurs with minimal contact by either the pups or the observers, with the mother, as well as frequent licking at the injection site. The symptoms of envenoming last for a few hours, creating a long period of separation between mothers and pups, which remained without maternal care.

In addition, mothers in group V2 did not collect the offspring during the observation period in the retrieval test. Retrieval is a common behavior displayed by rodents [38] and, usually, the assessment of maternal behavior is performed in the first week after delivery, as the offspring during this period are exclusively dependent on maternal care [15]. The time spent licking/grooming the pups decreases over the days, as does the time spent in contact with the pups. In the first 10 days after the delivery, the mothers stay longer in the nest and the pups grow, and maternal care tends to gradually decrease and the mother becomes less responsive towards her offspring [15,30]. Thus, the test was performed only in the V2 group, and was not performed in V10 and V16 groups.

Based on the above considerations, we believe that the stress of maternal deprivation could be responsible, at least in part, for the decrease in the convulsive threshold of the offspring.

However, we cannot disregard the possibility that, due to the physiological effects of the venom on the mother, some alteration occurs in the composition of the milk (milk components that could be in higher or lower concentrations), directly affecting the nervous system of the offspring, and studies are being developed in this regard in our laboratory.

It is also possible that some component of the venom or some cytokines produced by the mother are directly passed on, since scorpion accidents are capable of inducing an intense immune response in the injured individual. Several cytokines are increased in the plasma of envenomed patients, such as IL1-α, IL1-β, IL-6, IL-8, IL-10 TNF-α and IFN-γ [39,40,41]. In addition, it has been shown that scorpion venom can also alter some cytokines in pups of envenomed mothers [14].

These cytokines could affect the nervous system of the pups and be responsible for the decreased seizure threshold, as it is believed that inflammatory processes may be related to epileptogenesis [42]. Inflammation in neonates has already been shown to increase their susceptibility to seizures in adulthood and the induction of an inflammatory response was able to decrease the seizure threshold when performed between days 7 and 14 postnatally [43].

Evidence supports the hypothesis that cytokines not only act as inflammatory mediators, but also have neuromodulatory action. IL1-β is described as having excitatory effects in several brain regions [44]. In the hippocampus, TNF-α can increase the expression of AMPA receptors [45]. Concomitantly, this cytokine may be associated with decreased expression of GABA_A_ receptors [46]. Epidemiological analyses have demonstrated that central nervous system infections are a major cause of acquired epilepsy revised by [47]. It is due to changes in the physiological properties of neurons within the hippocampus [48].

Thus, the inflammatory process resulting from the envenoming could also be responsible for the decrease in the convulsive threshold of the offspring.

## 4. Conclusions

This study provides evidence of the interference caused by scorpion envenoming on maternal behavior and on the development of the central nervous system of the pups. A decrease in the care provided to immature pups is clear. Furthermore, it was possible to observe a lower seizure threshold in the offspring of injured mothers. Our results highlight the importance of the perinatal context in the individual’s health, even in adulthood.

## 5. Materials and Methods

### 5.1. Venom and Drugs

Dried venom of *T. serrulatus* obtained from the Strategic Nucleus of Venoms and Antivenoms of Butantan Institute (São Paulo, Brazil) was dissolved in 0.9% NaCl and injected (4.0 mg/kg, s.c.) into the back of lactating rats. The control group was injected with 0.9% NaCl (1.0 mL/kg s.c.).

Pentylenotetrazole (PTZ; Sigma-Aldrich™, St. Louis, MO, USA) was dissolved in 0.9% NaCl and injected in the adult offspring (initial dose of 20 mg/kg i.p., and booster doses of 10 mg/kg every 10 min).

### 5.2. Animals

Twelve male and twenty-four female Wistar rats (250–300 g), maintained under controlled conditions (food and water was permitted ad libitum, and the animals were maintained on a 12:12 light/dark schedule with lights on at 7 a.m.), were used for mating. All the experimental procedures were approved by our Institutional Ethics Committee for Experiments on Animals (No. 1927030818).

### 5.3. Animal Mating and Pregnancy Diagnosis

For mating, two females and one male were housed overnight. The impregnation was confirmed the next morning by the presence of spermatozoa in the vaginal smear. Pregnant females were housed individually until delivery.

### 5.4. Weaning

At weaning, the littermates were separated and housed by sex until 2 months of age, when the animals were submitted to the convulsive threshold test (two males and two females from each litter).

### 5.5. Experimental Groups

Lactating rats were divided into four groups (*n* = 6) that received the following treatments on post-natal (PN) day 2 (PN2), 10 (PN10) and PN16:

- CT group: 0.9% NaCl on PN 2, PN10, and PN16 (1 mL/kg, s.c.);
- V2 group: venom (4 mg/kg, s.c.) on PN2 and 0.9% NaCl on PN10, and PN16 (1 mL/kg, s.c.);
- V10 group: venom (4 mg/kg, s.c.) on PN10 and 0.9% NaCl on PN2, and PN16 (1 mL/kg, s.c.);
- V16 group: venom (4 mg/kg, s.c.) on PN16 and 0.9% NaCl on PN2, and PN10 (1 mL/kg, s.c.).

All the mothers were submitted to injections in PN2, PN10 and PN16, with 0.9% NaCl or venom according to the treatment group, so that all went through the same handling stress.

### 5.6. Evaluation of Maternal Behavior

Each female was provided with shredded paper one day before delivery for nest making. Maternal care was recorded with a digital camera (Canon Vixia HF R800, Tokyo, Japan) for 2 h after venom or 0.9% NaCl injection on PN2, PN10, and PN16. The time of arched-back nursing posture was recorded. Statistical analyses were performed by two-way ANOVA, and the level of significance was set at *p* < 0.001.

### 5.7. Retrieval Test

The same animals used in previous experiment were submitted to the retrieval test as described by [49]. Immediately after the maternal behavior observation session, the pups were removed from their mothers for 5 min. After this period, the whole litter was placed back in the housing cage in a dispersed manner. The mother was then observed for 10 min and the latency to pick up the first pup, the latency to pick up all the pups, and the number of pups picked up, were observed.

Statistical analyses were performed by two-way ANOVA, and the level of significance was set at *p* < 0.001.

### 5.8. Seizure Threshold

At 60 days of age, one male and one female from each litter received an initial dose of 20 mg/kg PTZ, and a booster of 10 mg/kg every 10 min until the occurrence of score 5 or more according to the score scale adapted from that proposed by [50] (Table 1).

The total injected volume of PTZ varied approximately between 0.6 mL (initial dose plus a booster) and 1.2 mL (initial dose plus three boosters).

After the observation, the average score in each experimental group was computed.

Statistical analyses were performed by the Kruskal–Wallis test, and the level of significance was set at *p* < 0.001 and *p* < 0.05.

## Figures and Tables

**Table 1:** Score scale adapted from that proposed by Fischer and Kittner (1998).

Score	Behavior
0	no evidence of convulsive activity
1	mouth and facial movements
2	myoclonic body jerking
3	jaw clonus
4	head and forelimb clonus
5	head and forelimb clonus with full rearing
6	head and forelimb clonus and falling
7	run with generalized tonic clonic convulsion
8	death

## Data Availability

Not applicable.
